# Biocompatibility of the Oxygenator on Pulsatile Flow by Electron Microscope

**DOI:** 10.21470/1678-9741-2021-0519

**Published:** 2023

**Authors:** Ahmet Tulga Ulus, Tuna Güray, Ece Ürpermez, Sertan Özyalçın, Ali Taner, Erdem Haberal, Mustafa Kocakulak

**Affiliations:** 1 Cardiovascular Surgery Clinic, Turkiye Yuksek Ihtisas Research and Training Hospital, Ankara, Turkey.; 2 Cardiovascular Surgery Department, Hacettepe University, Ankara, Turkey.; 3 Biomedical Engineering Department, Baskent University, Ankara, Turkey.; 4 Department of Cardiology, Aksaray Hospital, Aksaray, Turkey.; 5 Biomedical Engineering Department, İzmir Democracy University, Karabağlar, İzmir,Turkey.

**Keywords:** Pulsatile Flow, Oxygenators, Arterial Filter, Cardiopulmonary Bypass, Biocompatibility, Thoracic Surgery, Tumor Necrosis Factor-alpha

## Abstract

**Introduction:**

Extracorporeal perfusion flow type requires further investigation. The aim of this study is to compare the effects of pulsatile and nonpulsatile flow on oxygenator fibers that were analyzed by scanning electron microscope (SEM) and to extensively study patients’ coagulation profiles, inflammatory markers, and functional blood tests.

**Methods:**

Twelve patients who had open heart surgery were randomly divided into two groups; the nonpulsatile flow (group NP, six patients) and pulsatile flow (group P, six patients) groups. Both superficial view and axial sections of the oxygenator fiber samples were examined under SEM to compare the thickness of absorbed blood proteins and amount of blood cells on the surface of oxygenators. Platelet count, coagulation profile, and inflammatory predictors were also studied from the blood samples.

**Results:**

Fibrinogen levels after cardiopulmonary bypass were significantly lower in group NP (group P, 2.57±2.78 g/L; group NP; 2.39±0.70 g/L, P=0.03). Inflammatory biomarkers such as C-reactive protein, interleukin (IL)-6, IL-12, apelin, S100β, and tumor necrosis factor alpha were comparable in both groups. Axial sections of the oxygenator fiber samples had a mean thickness of 45.2 µm and 46.5 µm in groups P and NP, respectively, and this difference is statistically significant (P=0.006). Superficial view of the fiber samples showed obviously lower platelet, leukocyte, and erythrocyte levels in group P.

**Conclusion:**

Our study demonstrated that both cellular elements and protein adsorption on oxygenator fibers are lower in the group P than in the group NP. Pulsatile perfusion has better biocompatibility on extracorporeal circulation when analyzed by SEM technique.

## INTRODUCTION

Cardiopulmonary bypass (CPB) perfusion techniques can be divided into nonpulsatile and pulsatile perfusion. The pulsatile perfusion has higher force than the nonpulsatile flow, which can affect, in several ways, both the patient and oxygenator fibers^[1]^.

Some of the clinical and preclinical research reported beneficial impacts of the pulsatile perfusion mode of CPB. Pulsatile CPB is considered to be a more physiological flow than nonpulsatile flow. Enhanced microcirculation and diffusion, improved patency of the vascular bed, and low systemic vascular resistance are advantages of pulsatile CPB^[2]^.

CBP itself affects blood elements, causing thrombocytopenia, inadequate homeostasis, decrease in plasma coagulation factors, and platelet dysfunction, as described in previous studies^[3,4]^. CPB has been demonstrated to induce systemic inflammatory response^[5]^, affect the hematological system, and contribute to several postoperative complications^[2]^.

The effects of the perfusion type on oxygenator fibers are not extensively studied and hence not known. This study is focused on the effects of pulsatile and nonpulsatile flow on oxygenator fibers. The thickness of absorbed blood proteins and amount of blood cells on the surface of oxygenators were inspected with scanning electron microscope (SEM), and coagulation profiles, inflammatory markers, and functional blood tests of patients were extensively studied.

## METHODS

Twelve male patients, between 51 and 73 years of age, with normal left ventricular ejection fraction (LVEF), and scheduled for elective isolated coronary artery bypass grafting (CABG) were randomly divided into two groups. Nonpulsatile flow was used in group NP (six patients) and pulsatile flow in group P (six patients). The study was approved by the institutional authorization (11060-24072009). All patients signed a consent form.

Emergency procedures, patients with low LVEF (≤ 40%), patients who were receiving anticoagulants and antiplatelet medications for one week preceding their admission for surgery, and reoperations were excluded from the study.

Dideco Compactflo Evo oxygenator (Sorin, Sorin Group Italia, Mirandola, Italy), Jostra HL-20 roller pump (Jostra USA, Austin, Texas, United States of America), and Dideco D734 Micro 40 Adult (Sorin, Sorin Group Italia, Mirandola, Italy) arterial filter line were used. Prime solution was 1500 mL of Ringer’s solution and 200 mL of 20% mannitol. A perfusion flow of 2.2-2.42 L/min/m2 body surface area (BSA) was maintained during CPB. Pulse flow width 60%, base 25%, with a rate of 60 beats per minute were used in the pulsatile group.

Anesthetic regimens, CPB, and surgical procedures were standard in all cases. Anesthesia induction consisted of fentanyl, midazolam, and thiopentone sodium, and tracheal intubation was facilitated by pancuronium bromide. Heparin sulphate was used at an initial dose of 4 mg/kg followed by additional doses to maintain the activated clotting time > 400 seconds. The heart was arrested using Plegisol solution (Hospira Inc, North Chicago, United States of America), and arrest was maintained by using intermittent cold blood cardioplegia. The body temperature was monitored by rectal and blood probes throughout the surgery. All patients were operated under moderate hypothermic CPB. Patients were rewarmed up to 36,6°C of rectal temperature before discontinuation of CPB. Protamine sulphate was used in the same dose as the initial heparin dose. Coronary artery bypass technique was standardized method of anastomosis in all patients.

### Blood Analyses

Four blood samples were collected from the patients through the central venous line as scheduled - preoperatively, immediately after beginning of CPB, just after the CPB, and at the postoperative 24th hour. Hematocrit levels, platelet counts, coagulation profiles, serum creatinine, blood urea nitrogen, bilirubin, albumin, alanine aminotransferase (ALT), aspartate aminotransferase (AST), total protein amount, C-reactive protein (CRP), interleukin (IL)-6, IL-12, S100β, apelin, and tumor necrosis factor alpha (TNF-α) levels were analyzed and compared between both groups. IL-6, IL-12, and TNF-α were measured in duplicate using commercially available enzyme-linked immunosorbent assay (ELISA) kits (Diasource®, Nivelles, Belgium). Apelin levels were also measured in duplicate using commercially available ELISA kits (Phoneix Pharmaceuticals Inc®, California, United States of America). And S100β levels were measured in duplicate using commercially available ELISA kits (Diametra®, Milano, Italy).

### SEM Image Analyses

At the end of the operation, the oxygenator was filled with 2.5% glutaraldehyde solution with the help of roller pump. Glutaraldehyde solution is needed for the fixation of the blood elements and protein adsorption on the fibers. Oxygenator was cut by Dremel lithium-ion 800, and fibers were placed in 50-ml sterilized containers with 20 ml of saline. Each fiber was covered with 5 nm chromium by the Precision Etching and Coating System (PECS™) (Gatan 682, United States of America) and using sputter technique^[6]^.

Both superficial view and axial sections of the fiber samples were examined under SEM (FEI Quanta 200, Oregon, United States of America). SEM images were analyzed by using xT microscope Control software (Gatan Microscopy Suite, United States of America).

The sectional images of the fibers were obtained by cutting with ultramicrotome (Leica EM FC6, Germany). The fibers were frozen by pulverized 50% propanol alcohol and 50% water at -120 °C. Nitrogen gas was sprayed on fibers in order to vaporize the water and alcohol on them following the cutting procedure. All samples were placed on silicon wafer and analyzed with SEM.

The SPSS Inc. Released 2007, SPSS for Windows, version 16.0, Chicago, SPSS Inc. was used. Descriptive data were expressed as mean and standard deviation. Parametric tests were used for data with a normal distribution, and nonparametric tests were applied to data without normal distribution. Distribution of normality was tested with the Kolmogorov-Smirnov test. The Mann-Whitney U and Wilcoxon tests were used to compare variables between groups. Chi-squared, Fisher’s, and Mantel-Haenszel tests were performed for comparison of categorical variables. Level of significance was set at *P*<0.05.

## RESULTS

Mean age, body weight, BSA, CPB time, pump flow, minimum temperatures, aortic cross-clamping time, and number of target vessels were compared between both groups, and there was no significant difference between them (Table 1).

**Table 1:** Clinical data and cardiopulmonary perfusion findings.

	Group P (n=6)	Group NP (n=6)
Age (years)	66.0±8.5	59.7±8.0
Weight (kg)	84.8±7.1	76.4±9.7
BSA (m^2^)	1.9±0.03	1.8±0.11
CPB time (min)	112.6±30.7	101.1±26.6
Cross-clamping time (min)	66.6±19.5	62.2±21.3
Pump flow (L/min/)	4.6±0.2	4.5±0.2
Number of target vessels	3.0±0.8	3.2±0.7
Minimum CPB temperature (°C)	30.4±0.5	30.7±1.8

BSA=body surface area; CPB=cardiopulmonary bypass; NP=nonpulsatile; P=pulsatile

Hematocrit values, platelet counts, and fibrinogen and prothrombin levels decreased during and after CPB in both groups, and there was no significant difference between the groups; only a fibrinogen measurement after CPB had significantly lower level in group NP (group P, 2.57±2.78 g/L; group NP, 2.39±0.70 g/L; *P*=0.03) (Table 2). D-dimer levels increased during CPB and returned to preoperative levels after 24 hours following CPB (Table 2). Blood urea nitrogen, creatinine, total protein, albumin, total bilirubin, and ALT levels were similar in both groups in all measurement intervals. ALT levels were significantly lower in group P preoperatively and during and after CPB (Table 3). Inflammatory biomarkers such as CRP, IL-6, IL-12, apelin, S100β, and TNF-α were comparable in both groups. There was no significant difference between groups at any time point, although all increased during the follow-up (Table 4).

**Table 2:** Hematologic and coagulation profile of the patients according to the groups.

	Group P (n=6)	Group NP (n=6)	*P*-value
**Hematocrit values**			
Preoperative	35.4±3.4	36.4±4.6	0.68
During CPB	27.1±4.3	26.5±5.4	0.84
After CPB	28.2±4.2	28.9±4.1	0.79
Postoperative 24^th^ hour	27.4±0.1	30.4±2.4	0.35
**Platelet values (n/mL)**			
Preoperative	138.2±25.0	191.3±28.3	0.68
During CPB	142.6±22.2	184.7±59.6	0.14
After CPB	119.2±43.1	172.2±49.9	0.06
Postoperative 24^th^ hour	150.0±0.1	194.2±44.1	0.43
**Fibrinogen (g/L)**			
Preoperative	3.42±0.72	3.02±0.78	0.47
During CPB	2.93±0.40	2.59±1.23	0.14
After CPB	2.57±2.78	2.39±0.70	0.03
Postoperative 24^th^ hour	2.90±0.01	4.47±1.01	0.06
**D-Dimer (mcg/mL)**			
Preoperative	1.61±2.63	1.16±2.02	0.42
During CPB	5.98±1.97	5.43±3.44	0.12
After CPB	8.33±1.02	6.63±4.77	0.20
Postoperative 24^th^ hour	1.21±0.30	1.00±0.39	0.27
**Prothrombin (U/ml)**			
Preoperative	737±374	632±343	0.91
During CPB	604±259	515±168	0.29
After CPB	517±222	535±138	0.29
Postoperative 24^th^ hour	454±117	530±101	0.26

CPB=cardiopulmonary bypass; NP=nonpulsatile; P=pulsatile

**Table 3:** Liver and kidney functions of the patients according to the groups.

	Group P (n=6)	Group NP (n=6)	*P*-value
**Creatinine (mg/dl)**			
Preoperative	0.96±0.11	0.84±0.17	0.41
During CPB	0.83±0.17	0.79±0.39	0.64
After CPB	0.92±0.12	0.73±0.25	0.31
Postoperative 24^th^ hour	0.78±0.01	0.92±0.22	0.90
**Blood urea nitrogen (mg/dl)**			
Preoperative	33.5±12.5	36.4±10.5	0.78
During CPB	35.6±9.4	34.8±8.9	0.83
After CPB	32.5±8.5	34.2±9.3	0.66
Postoperative 24^th^ hour	38.0±0.1	32.2±2.5	0.90
**Bilirubin (total) (mg/dl)**			
Preoperative	0.5±0.3	0.6±0.7	0.62
During CPB	0.3±0.2	0.6±0.7	0.35
After CPB	0.4±0.2	0.6±0.5	0.49
Postoperative 24^th^ hour	2.5±2.1	2.2±2.4	0.12
**AST (IU/L)**			
Preoperative	13.3±3.0	21.1±11.9	0.12
During CPB	22.5±3.6	25.6±9.6	0.13
After CPB	24.6±3.2	37.6±20.2	0.08
Postoperative 24^th^ hour	41.0±0.2	30.7±7.3	0.1
**ALT (IU/L)**			
Preoperative	13.6±0.5	25.7±19.6	0.01
During CPB	10.7±1.2	20.4±15.8	0.03
After CPB	10.3±2.3	23.0±13.6	0.04
Postoperative 24^th^ hour	14.5±6.3	21.0±2.8	0.05
**Protein (total) (mg/dl)**			
Preoperative	5.9±0.3	5.4±1.8	0.32
During CPB	3.7±0.3	3.9±0.8	0.32
After CPB	3.7±0.4	3.0±1.0	0.31
Postoperative 24^th^ hour	5±0.2	5.4±0.6	0.50
**Albumin (mg/dl)**			
Preoperative	3.6±0.2	3.6±0.6	0.52
During CPB	2.1±0.2	2.5±0.9	0.15
After CPB	2.2±0.2	2.3±0.6	0.45
Postoperative 24^th^ hour	3.3±0.3	3.3±0.4	0.50

ALT=alanine aminotransferase; AST=aspartate aminotransferase; CPB=cardiopulmonary bypass; NP=nonpulsatile; P=pulsatile

**Table 4:** Inflammatory markers of the patients according to the groups.

	Group P (n=6)	Group NP (n=6)	*P*-value
**CRP (mg/dl)**			
Preoperative	8.2±8.2	5.8±5.5	0.07
During CPB	4.3±4.4	4.9±4.5	0.82
After CPB	5.0±4.5	3.6±0.8	0.28
Postoperative 24^th^ hour	75.6±7.3	74.6±5.0	0.92
**Apelin (ng/ml)**			
Preoperative	1069±137	1217±229	0.49
During CPB	1588±216	1438±274	0.31
After CPB	1480±61	1378±215	0.25
Postoperative 24^th^ hour	1180±315	1286±251	0.65
**IL-6 (pg/ml)**			
Preoperative	37.0±6.0	38.1±8.6	0.12
During CPB	136.3±67.4	245.3±366.0	0.13
After CPB	334±147	303±489	0.40
Postoperative 24^th^ hour	310±180	85.2±41.6	0.30
**IL-12 (pg/ml)**			
Preoperative	344±173	303±126	0.71
During CPB	384±100	352±182	0.32
After CPB	432±151	314±131	0.87
Postoperative 24^th^ hour	228±156	238±172	0.47
**S100β (pg/ml)**			
Preoperative	39.6±2.5	52.3±27.2	0.25
During CPB	206±43	128±44	0.88
After CPB	224±110	187±57	0.11
Postoperative 24^th^ hour	98±45	94±102	0.33
**TNF-α (pg/ml)**			
Preoperative	43±10.4	46.8±17.6	0.42
During CPB	38.3±25.3	64.2±85.3	0.32
After CPB	66.3±31.1	90.3±67.3	0.17
Postoperative 24^th^ hour	49.0±28.1	46.7±19.0	0.48

CPB=cardiopulmonary bypass; CRP=C-reactive protein; IL=interleukin; NP=nonpulsatile; P=pulsatile; TNF-α=tumor necrosis factor alpha

**Fig. 1:**
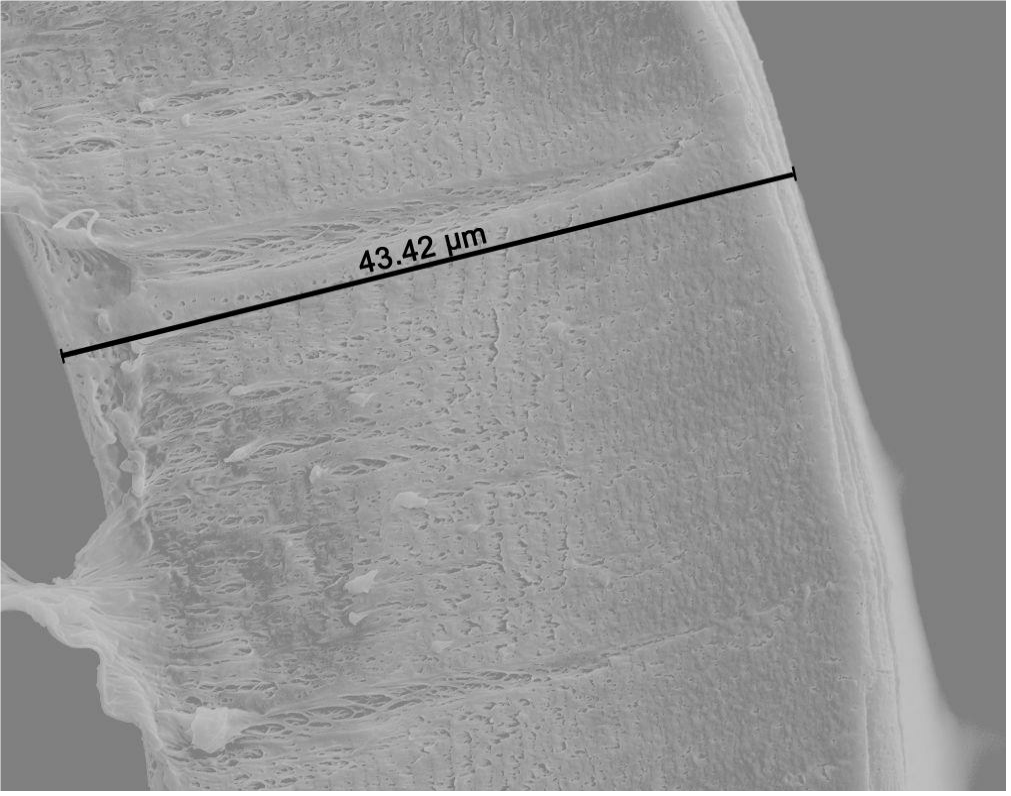
Section of non-used oxygenator fiber axis view by scanning electron microscope (voltage 10.00 kV, sample-objective lens distance 16.8 mm, and magnification 5000×). Fiber thickness is 43.4 µm before cardiopulmonary bypass perfusion (reference value).

**Fig. 2:**
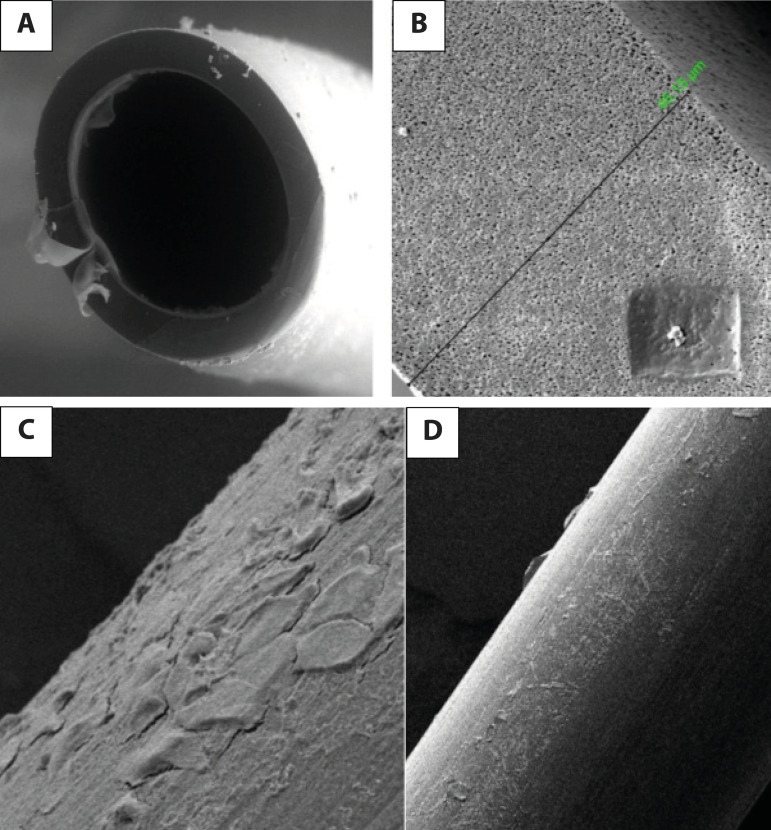
A. Section of a fiber axis view by scanning electron microscope (voltage 5.00 kV, sample-objective lens distance 20.1 mm, and magnification 500×) with an oxygenator following nonpulsatile cardiopulmonary bypass (CPB) flow. B. A sample of fiber thickness is 46.15 µm measured following nonpulsatile CPB perfusion (voltage 5.00 kV, sample-objective lens distance 20.1 mm, and magnification 5000×). C. Surface of a fiber axis view by scanning electron microscope (voltage 5.00 kV, sample-objective lens distance 11.6 mm, and magnification 4000×) with an oxygenator following nonpulsatile CPB flow. D. Surface of a fiber axis view by scanning electron microscopy (voltage 5.00 kV, sample-objective lens distance 11.3 mm and magnification 500x) with an oxygenator following pulse CPB flow.

**Fig. 3:**
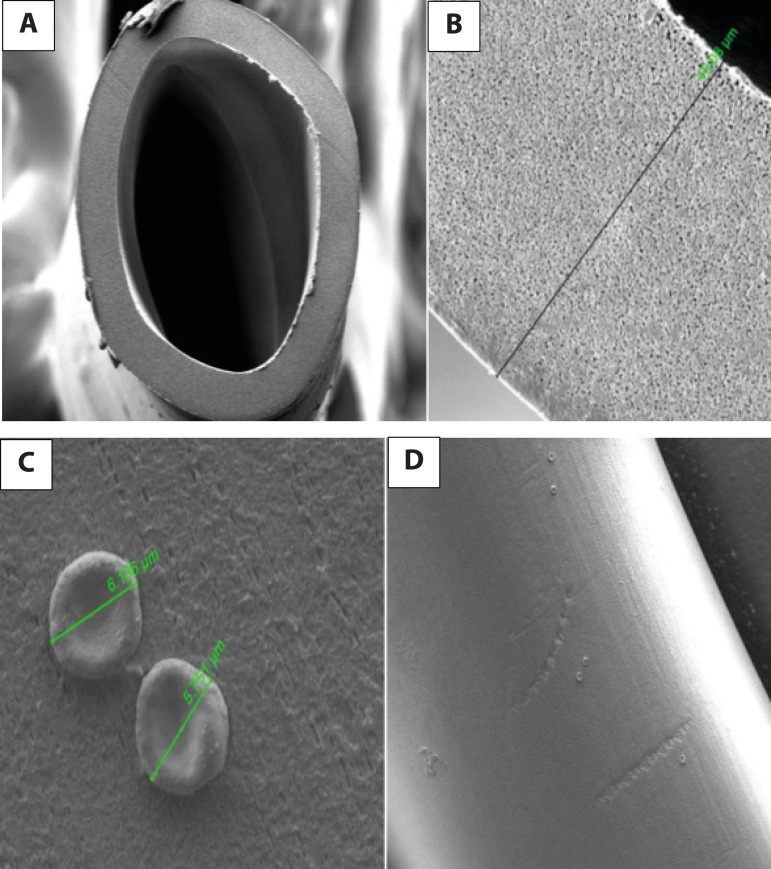
A. Section of a fiber axis view by scanning electron microscope (voltage 5.00 kV, sample-objective lens distance 20.1 mm, and magnification 500×) with an oxygenator following pulsatile cardiopulmonary bypass (CPB) flow. B. A sample of fiber thickness is 45.08 µm measured following pulsatile CPB perfusion (voltage 5.00 kV, sample-objective lens distance 20.1 mm, and magnification 5000×). C. Surface of a fiber axis view by scanning electron microscope (voltage 2.00 kV, sample-objective lens distance 8.7 mm, and magnification 10000×) with an oxygenator following pulsatile CPB flow. D. Surface of a fiber axis view by scanning electron microscope (voltage 2.00 kV, sample-objective lens distance 8.7 mm, and magnification 600×) with an oxygenator following pulsatile CPB flow.

**Fig. 4:**
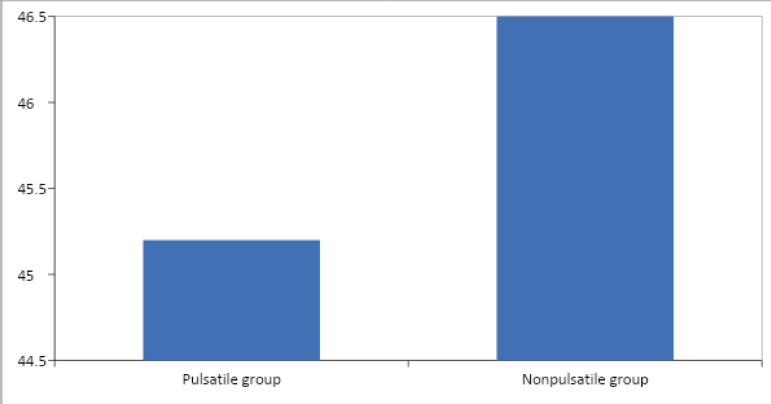
The amount of protein adsorbed on the oxygenator fiber (P=0.006) (reference=43.4 µm).

Axial sections of the fiber samples which were examined under SEM were analyzed. Section of the fibers before and after CPB were measured to understand the adsorbed protein thickness on fibers. Section of fiber axis before CPB (non-used oxygenator) was measured as 43.4 µm and it was accepted as reference value (Figure 1). Samples of fiber thickness measurements are shown in Figures 2A, 2B and 3A, 3B, following nonpulsatile and pulsatile CPB perfusion. The mean fiber thickness from the axial images were calculated as 45.2 µm and 46.5 µm in groups P and NP, respectively (Figure 4). The difference is statistically significant (*P*=0.006).

Superficial view of the fiber samples which were examined under SEM were subjectively analyzed from the images. It is obvious that the platelet, leukocyte, and erythrocyte amount is lower in group P than ingroup NP (Figure 2C, 2D and Figure 3C and 3D).

## DISCUSSION

Although research on pulsatile flow have long been performed, it is not reached a clear conclusion on the best technique for CPB flow. A mechanical system for pulsatile CPB was also presented, which has advantages as simplicity, low cost, and the synchronization of pulse generation with the arterial roller^[7]^. None of the studies have investigated SEM analysis of oxygenator fibers and patients’ blood tests, including inflammatory cytokines, coagulation, and hematological analysis, with pulsatile perfusion.

The oxygenator is the most important part of extracorporeal circulation. Blood and oxygenator-fiber interactions during the perfusion were not completely searched. This is where oxygen exchange occurs, and the detrimental effects to the blood elements mostly happen on the membrane surfaces^[8]^. Protein adsorption begins in seconds on biomaterials^[9]^. Tanaka M. demonstrated the adsorption of the plasma proteins on the surface of the oxygenator, which is an important drawback of extracorporeal circulation^[10]^. When those proteins were sticked to the surface, the structure of the surface changes and platelets begin activate and adhere to the surface of the oxygenator^[11]^.

Although many studies revealed the superiority of the pulsatile perfusion on tissue metabolism, cell diffusion, and microcirculation, it is not widely used during open-heart surgery^[12]^. We compared both techniques using clinical outputs and SEM analysis. Undar A. is studying pulsatile flow circulation for long years and uses energy equivalent pressure (area beneath the hemodynamic power curve and area beneath the pump flow of the pulse cycle) and surplus hemodynamic energy to measure pulsatility. The controversy over pulsatile CPB could be the real quantification and precise evaluation of the method^[13]^. Vieira F.U. mentioned the comparisons by using blood tests to verify the influence of changes in hemolysis involving pulsatile/nonpulsatile pumps^[14]^.

In our study, hematocrit value, platelet counts, fibrinogen, and prothrombin levels decreased in both groups insignificantly. Fibrinogen level was significantly low in group NP following CPB. The decrease in platelet count persisted until 24 hours postoperatively. Laufer et al.^[3]^ observed normalization of platelet counts several days after CPB. Platelet count changes have been attributed to the formation of platelet aggregates on the oxygenator^[4]^. Although it is thought that pulsatile flow causes mechanical disruption of platelets and is responsible for a decrease in platelet count, there is no significant difference between the groups related with hematocrit value, platelet counts, fibrinogen, and prothrombin levels in our study. Higher fibrinogen level in group P shows that it was not exhausted during the CPB. D-dimer levels increased during the CPB flow and returned to normal values because of the possible pump effect. Agirbasli M.A. studied the components of the fibrinolytic system, like tissue plasminogen activator and plasminogen activator inhibitor-1 in 40 children. He concluded that pulsatile perfusion is beneficial for endogenous fibrinolytic system^[15]^.

Blood urea nitrogen, creatinine, total protein, albumin, total bilirubin, and ALT levels were comparable in both CPB flow groups. Only ALT levels were significantly lower in group P. Many studies shown that pulsatile perfusion decreases peripheral vascular resistance and enhances tissue perfusion^[5,6]^. Olinger et al.^[16]^ reported that pulsatile flow also preserves renal functions. Jiang Q. concluded that S100β, urinary neuron-specific enolase, and plasma β2-microglobulin level were significantly increased at six and 24 hours after surgery in two groups and were significantly higher in the nonpulsatile group. The pulsatile energy influences the secretion of endothelial and inflammatory factors and demonstrate better cerebral and kidney protective effect at the biological marker level^[17]^.

On the other hand, Abramov D. could not find any benefit of pulsatile flow over nonpulsatile perfusion in a study of 1,820 CABG patients. Neither overall mortality/morbidity reduced, nor renal dysfunction^[18]^. Also, Alghamdi A.A. searched MEDLINE®, Embase®, and the Cochrane controlled trial registries (or CCTR) on the Cochrane library and defined that there is not supportive data for or against pulsatile perfusion to reduce mortality, myocardial infarction, stroke, or renal insufficiency^[18]^.

During CPB, release of cytokines have been documented recently by several studies^[19]^. TNF-α is a proinflammatory cytokine that induce hypotension and organ damage. IL-6 has an important role in the acute phase of inflammatory response and is directly responsible for the postoperative complications^[19]^. Apelin signaling effectively suppresses the inflammation factors such as TNF-α and IL^[20]^. It also increases the vasodilatation and peripheral vascular resistance^[21]^. S100β proteins are used for the neurological follow-up^[22]^. Studies showed that reduced IL-12 serum levels were correlated with the incidence of postoperative complications following cardiac surgery^[23]^. Sezai A. showed a reduced cytokine activity, endothelial damage, renal function, and pulmonary function on pulsatile perfusion^[24,25]^. In our study, inflammatory biomarkers such as CRP, IL-6, IL-12, apelin, S100β, and TNF-α were all increased during the CPB, but the levels were comparable in both groups. There were no significant differences between the groups at any time point. The results of the present study were comparable with the others as cytokines rose after CPB in both groups and declined thereafter.

The biocompatibility of the oxygenator according to the flow type was evaluated by SEM analyses. Detailed analyses of the oxygenators, regarding the protein adsorption, and superficial image analysis, in order to view the cellular deposition, were not performed till now on pulsatile CPB. Axial sections of the fiber samples were measured on non-used oxygenator to obtain reference value by SEM. We found that the protein adsorption thickness on oxygenator fibers was affected by the flow type. The difference between the reference value and thickness measurement following CPB on fibers was calculated as protein adsorption value. This value was 1.8 µm in group P and 3.1 µm in group NP. The difference is 1.3 µm and is statistically significant. The protein adsorption on oxygenator fibers is extremely low on pulsatile perfusion flow during the CPB. Superficial view of fiber samples was also analyzed using SEM. Although the analysis is qualitative, it is clear that the platelet, leukocyte, and erythrocyte amount are lower ingroup P than in group NP. The pulsatile group had higher blood fibrinogen level which also means lower adhesion. Superficial surface examination and protein adsorption of the fibers also prove this relation.

### Limitations

As a limitation of the study, we could not describe the quantification of pulsatile perfusion in terms of energy equivalent pressure and surplus hemodynamic energy.

## CONCLUSION

In conclusion, the inflammatory biomarkers increased during CPB. It was demonstrated that both cellular elements and protein adsorption on oxygenator fibers are lower in pulsatile perfusion group. The platelet, leukocyte, and erythrocyte amount are also lower in the pulsatile flow group. Pulsatile perfusion has better biocompatibility on extracorporeal circulation when analyzed by SEM technique.

## References

[1] Taylor KM, Longmore DB (1981). Towards safer cardiac surgery.

[2] Chiu IS, Chu SH, Hung CR (1984). Pulsatile flow during routine cardiopulmonary
bypass. J Cardiovasc Surg (Torino).

[3] Laufer N, Merin G, Grover NB, Pessachowicz B, Borman JB (1975). The influence of cardiopulmonary bypass on the size of human
platelets. J Thorac Cardiovasc Surg.

[4] de Leval M, Hill JD, Mielke H, Bramson ML, Smith C, Gerbode F (1972). Platelet kinetics during extracorporeal
circulation. Trans Am Soc Artif Intern Organs.

[5] Philbin DM, Levine FH, Emerson CW, Coggins CH, Buckley MJ, Austen WG (1979). Plasma vasopressin levels and urinary flow during cardiopulmonary
bypass in patients with valvular heart disease: effect of pulsatile
flow. J Thorac Cardiovasc Surg.

[6] Shah PJ, Wu Z, Sarangan AM (2013). Effects of CO2 critical point drying on nanostructured SiO2 thin
films after liquid exposure. Thin solid films.

[7] dos Reis CL, Barbosa Evora PR, de Freitas Ribeiro PJ, Brasil JC, Garcia Otaviano A, Lisboa Bongiovani H (1987). A simple mechanical system for pulsatile cardiopulmonary
bypass. J Cardiovasc Surg (Torino).

[8] Iwahashi H, Yuri K, Nosé Y (2004). Development of the oxygenator: past, present, and
future. J Artif Organs.

[9] Gorbet MB, Sefton MV (2004). Biomaterial-associated thrombosis: roles of coagulation factors,
complement, platelets and leukocytes. Biomaterials.

[10] Tanaka M, Motomura T, Kawada M, Anzai T, Kasori Y, Shiroya T (2000). Blood compatible aspects of poly(2-methoxyethylacrylate)
(PMEA)--relationship between protein adsorption and platelet adhesion on
PMEA surface. Biomaterials.

[11] Rabe M, Verdes D, Seeger S (2011). Understanding protein adsorption phenomena at solid
surfaces. Adv Colloid Interface Sci.

[12] Kocakulak M, Aşkin G, Kuçukaksu S, Tarcan O, Pişkin E (2005). Pulsatile flow improves renal function in high-risk cardiac
operations. Blood Purif.

[13] Undar A (2003). Energy equivalent pressure formula is for precise quantification
of different perfusion modes. Ann Thorac Surg.

[14] Undar A, Ji B, Lukic B, Zapanta CM, Kunselman AR, Reibson JD (2006). Quantification of perfusion modes in terms of surplus hemodynamic
energy levels in a simulated pediatric CPB model. ASAIO J.

[15] Vieira FU Jr, Vieira RW, Antunes N, Petrucci O, Oliveira PP, Serra MM (2009). Analysis of the hydrodynamic profile in different roller pumps
models used in cardiopulmonary bypass. Rev Bras Cir Cardiovasc.

[16] Aĝirbaşli MA, Song J, Lei F, Wang S, Kunselman AR, Clark JB (2014). Comparative effects of pulsatile and nonpulsatile flow on plasma
fibrinolytic balance in pediatric patients undergoing cardiopulmonary
bypass. Artif Organs.

[17] Olinger GN, Hutchinson LD, Bonchek LI (1983). Pulsatile cardiopulmonary bypass for patients with renal
insufficiency. Thorax.

[18] Jiang Q, Sun J, Xu L, Chang X, Sun L, Zhen Y (2021). Frequency domain analysis and clinical outcomes of pulsatile and
non-pulsatile blood flow energy during cardiopulmonary
bypass. Perfusion.

[19] Steinberg JB, Kapelanski DP, Olson JD, Weiler JM (1993). Cytokine and complement levels in patients undergoing
cardiopulmonary bypass. J Thorac Cardiovasc Surg.

[20] Butler J, Parker D, Pillai R, Westaby S, Shale DJ, Rocker GM (1993). Effect of cardiopulmonary bypass on systemic release of
neutrophil elastase and tumor necrosis factor. J Thorac Cardiovasc Surg.

[21] Frering B, Philip I, Dehoux M, Rolland C, Langlois JM, Desmonts JM (1994). Circulating cytokines in patients undergoing normothermic
cardiopulmonary bypass. J Thorac Cardiovasc Surg.

[22] Chen MM, Ashley EA, Deng DX, Tsalenko A, Deng A, Tabibiazar R (2003). Novel role for the potent endogenous inotrope apelin in human
cardiac dysfunction. Circulation.

[23] Japp AG, Cruden NL, Barnes G, van Gemeren N, Mathews J, Adamson J (2010). Acute cardiovascular effects of apelin in humans: potential role
in patients with chronic heart failure. Circulation.

[24] Sezai A, Shiono M, Nakata K, Hata M, Iida M, Saito A (2005). Effects of pulsatile CPB on interleukin-8 and endothelin-1
levels. Artif Organs.

[25] Kocakulak M, Koçum İC, Ayhan H (2012). Investigation of inflammatory response at blood-poly
(2-methoxyethyl acrylate) (PMEA) interface in vivo via scanning tunneling
microscope. Journal of bioactive and compatible polymers.

